# Supplementary material for: Heterogeneous appetite patterns in depression: computational modeling of nutritional interoception, reward processing, and decision-making
Source: Front Hum Neurosci. 2024 Dec 16;18:1502508. doi: 10.3389/fnhum.2024.1502508 (PMC11683075; doi:10.3389/fnhum.2024.1502508)
Supplement: Supplementary file 2 [file Table_2.docx]

Supplementary Table 2; Mountain-climbing task

| Free parameter | Value | Explanation |
| --- | --- | --- |
| $\alpha^{Q}$ | 0.3 | Learning rate of state-action values |
| $\beta$ | 0.5 | Inverse temperature of action selections |
| $\gamma$ | 0.9 | Discount rate |
| $m$ | 3 | Free parameter of the drive function |
| $n$ | 4 | Free parameter of the drive function |
| $\tau$ | 200 | Attenuation rate of the internal state |
| $\alpha^{\hat{K}}$ | 0.3 | Learning rate of predicted value of increases in the internal state |
| $K_{small}$ | 0.05 | Volume of small intake |
| $K_{large}$ | 15 | Volume of large intake |
| $s$ | 2 | Cost to climb |
| $H^{*}$ | 200 | The ideal internal state |
| $H_{0}$ | 100 | The depleted initial internal state |
| $\iota$ | 1 | Degree of interoception |
| $\iota_{high}$ | 1.01 | Overestimated interoception |
| $\iota_{low}$ | 0.7 | Underestimated interoception |
| $\gamma_{high}$ | 0.99 | High-gamma |
| $\gamma_{low}$ | 0 | Low-gamma |
| $\beta_{high}$ | 0.63 | High-beta |
| $\beta_{low}$ | 0.54 | Low-beta |
