# Supplementary figures and images for: Heterogeneous appetite patterns in depression: computational modeling of nutritional interoception, reward processing, and decision-making
Source: Front Hum Neurosci. 2024 Dec 16;18:1502508. doi: 10.3389/fnhum.2024.1502508 (PMC11683075; doi:10.3389/fnhum.2024.1502508)

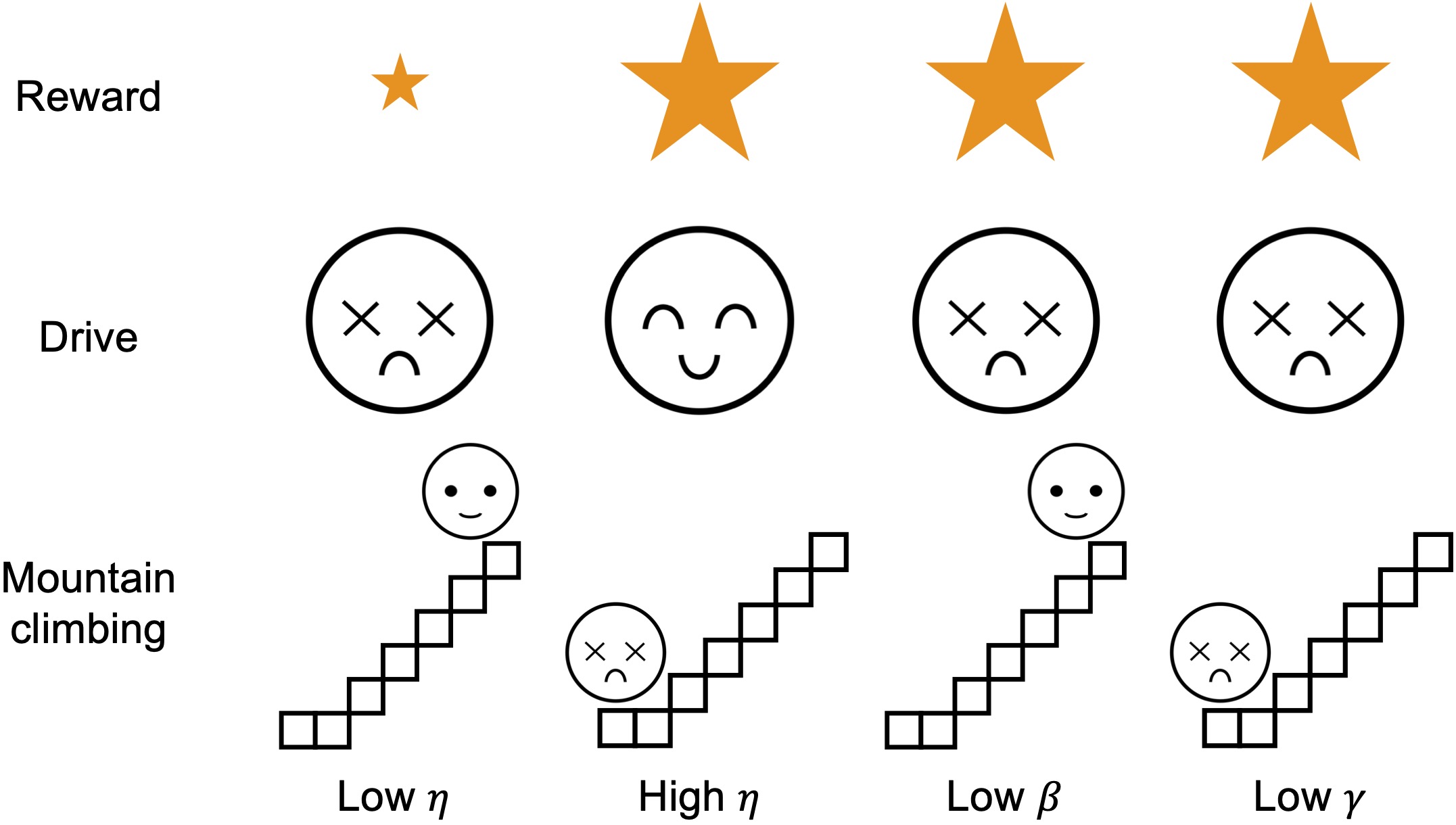

Supplement: Supplementary file 3 [file Image_1.jpeg]
